# Pharmacokinetic Interaction Between Olaparib and Regorafenib in an Animal Model

**DOI:** 10.3390/pharmaceutics16121575

**Published:** 2024-12-09

**Authors:** Danuta Szkutnik-Fiedler, Agnieszka Karbownik, Filip Otto, Julia Maciejewska, Alicja Kuźnik, Tomasz Grabowski, Anna Wolc, Edmund Grześkowiak, Joanna Stanisławiak-Rudowicz, Edyta Szałek

**Affiliations:** 1 Department of Clinical Pharmacy and Biopharmacy, Poznań University of Medical Sciences, Rokietnicka 3, 60-806 Poznań, Poland; akarbownik@ump.edu.pl (A.K.); f.otto@ump.edu.pl (F.O.); grzesko@ump.edu.pl (E.G.); jstanislawiak@ump.edu.pl (J.S.-R.); eszalek@ump.edu.pl (E.S.); 2 Students Scientific Association at the Department of Clinical Pharmacy and Biopharmacy, Faculty of Pharmacy, Poznań University of Medical Sciences, Rokietnicka 3, 60-806 Poznań, Poland; julia.maciejewska1409@gmail.com (J.M.); alicjakuznik00@gmail.com (A.K.); 3 Department of Inorganic Chemistry, Faculty of Pharmacy, Medical University of Gdańsk, M. Skłodowskiej-Curie 3a, 80-210 Gdańsk, Poland; tomasz.grabowski@gumed.edu.pl; 4 Department of Animal Science, Iowa State University, 239E Kildee Hall, Ames, IA 50011, USA; awolc@iastate.edu; 5 Hy-Line International, 2583 240th Street, Dallas Center, IA 50063, USA; 6 University Clinical Hospital, Szamarzewskiego 84/86, 60-569 Poznań, Poland

**Keywords:** drug–drug interaction, pharmacokinetics, olaparib, regorafenib, rats

## Abstract

Background: Olaparib (OLA) and regorafenib (REG) are metabolized by the CYP3A4 isoenzyme of cytochrome P450. Both drugs are also substrates and inhibitors of the membrane transporters P-glycoprotein and BCRP. Therefore, the potential concomitant use of OLA and REG may result in clinically relevant drug–drug interactions. Knowledge of the influence of membrane transporters and cytochrome P450 enzymes on the pharmacokinetics of drugs makes it possible to assess their impact on the efficacy and safety of therapy. Purpose: The study aimed to evaluate the bilateral pharmacokinetic interactions of OLA and REG and its active metabolites after a single administration in healthy rats. Methods: The study was performed in male Wistar rats (n = 24) randomly divided into three groups: one study group, I_REG+OLA_ (n = 8), received REG with OLA, and two control groups, II_REG_ (n = 8) and III_OLA_ (n = 8), received REG and OLA, respectively. The concentrations of OLA, REG, REG-N-oxide (M-2), and N-desmethyl-REG-N-oxide (M-5) were determined by ultra-performance liquid chromatography–tandem mass spectrometry (UPLC-MS/MS). The values of the pharmacokinetic parameters of OLA, REG, M-2, and M-5 were determined by non-compartmental analysis with linear interpolation. Results: After OLA administration, the pharmacokinetic parameters of REG (AUC_0–∞_, t_max_, and t_0.5_) increased significantly by 3.38-, 2.66-, and 1.82-fold, respectively. On the other hand, REG elimination parameters, i.e., k_el_ and Cl/F, were significantly reduced in the study group by 1.77- and 1.70-fold, respectively. In the study group, C_max_ and AUC_0–t_ values were also 7.22- and 8.86-fold higher for M-2 and 16.32- and 17.83-fold higher for M-5, respectively. The Metabolite M-2/Parent and Metabolite M-5/Parent ratios for C_max_ and AUC_0–t_ increased by 6.52-, 10.74-, 28-, and 13-fold, respectively. After administration of OLA with REG, the C_max_, AUC_0–t_, and AUC_0–∞_ of OLA increased by 2.0-, 3.4-, and 3.4-fold, respectively, compared to the control group. Meanwhile, Cl/F and Vd/F of OLA were significantly decreased in the presence of REG. Conclusions: OLA was shown to significantly affect the pharmacokinetics of REG and its active metabolites M-2 and M-5 in rats after co-administration of both drugs. There was also a significant effect of REG on the pharmacokinetics of OLA, which may have clinical relevance. The AUC ratios (study group/control group) were 3.41 and 3.39 for REG and OLA, respectively, indicating that REG and OLA were moderate inhibitors in this preclinical study. The results obtained need to be confirmed in clinical studies. This study may provide guidance on the safety of using both drugs in clinical practice.

## 1. Introduction

Cancer patients are a group particularly susceptible to drug–drug interactions (DDIs) due to polypharmacotherapy. Serious consequences and clinical implications can result from pharmacodynamic interactions, but also from changes in drug pharmacokinetics [1,2,3]. Among the most commonly diagnosed cancers in both men and women are cancers of the gastrointestinal tract, including colorectal cancer (CRC) [4,5].

One of the drugs used in CRC is regorafenib (REG), which is recommended for patients with metastatic colorectal cancer (CRC), including those who have failed treatment with fluoropyrimidine-based chemotherapy or treatment with an anti-VEGF or anti-EGFR agent. REG is also indicated in patients with unresectable or metastatic submucosal gastrointestinal cancer who have progressed or are intolerant to prior treatment with imatinib and sunitinib, and in patients with hepatocellular carcinoma who have received prior treatment with sorafenib [6,7,8].

REG targets several protein kinases involved in tumor angiogenesis, oncogenesis, and metastasis. The drug has broad pharmacological activity, blocking the activity of vascular endothelial growth factor (VEGF), angiopoietin (TIE2), fibroblast growth factor (FGFR), platelet-derived growth factor (PDGFR), and the RET oncogene [6,7,8,9,10].

Preclinical studies confirm that inhibitors of poly-ADP-ribose polymerases (PARP-1, PARP-2, and PARP-3) may also be helpful in the treatment of CRC and other cancers associated with less common gene mutations such as ATM, CHECK2, PALB2, and RAD51 [11,12,13]. PARP inhibition alone is thought to be cytostatic but not cytotoxic against ATM-deficient tumor cells, so a combination of a PARP inhibitor and another chemotherapeutic agent is required to induce cell death [11,12,13,14].

An example of a PARP inhibitor that has been shown to have such an effect is olaparib (OLA) [11,12,15], which is approved for the maintenance treatment of breast cancer, platinum-sensitive ovarian cancer, pancreatic cancer, prostate cancer in patients with inherited mutations in the BRCA1 or BRCA2 genes [15], and endometrial cancer [16].

OLA has also been shown to be well-tolerated and highly effective in prolonging survival in patients with advanced hepatocellular carcinoma (HCC) in combination with other anticancer agents [17,18,19,20,21,22]. This is important information regarding new therapeutic options, as the median overall survival for patients with gastrointestinal cancers is only 1 year on average. In addition, only 3% of patients with stage IV disease at diagnosis achieve 5-year progression-free survival [23].

The combination of OLA with various kinase inhibitors [24,25,26,27,28,29,30,31,32] shows promising results in enhancing its efficacy and overcoming resistance in different cancer types. These combinations were generally well-tolerated and demonstrated significant clinical activity, warranting further investigation and potential clinical application.

In humans, both OLA [15,22,32] and REG [6,7,8,9,10] are metabolized by the cytochrome P450 isoenzyme CYP3A4, and REG is additionally metabolized by the uridine diphospho-glucuronotransferase UGT1A9. In addition to being a CYP3A4 substrate, OLA may also inhibit the activity of this isoenzyme in vitro. A similar effect may be expected under in vivo conditions. OLA [15,22,32] and REG, as well as the REG metabolites M-2 and M-5 [6,7,8,9,10], are substrates of the membrane transporter P-glycoprotein (P-gp). In addition, both drugs have P-gp inhibitory activity [6,7,8,9,10,15,22,32].

REG and its active metabolites M-2 and M-5 are also substrates of the breast cancer resistance protein (BCRP) membrane transporter, and REG itself is an inhibitor of BCRP. OLA also has inhibitory activity against BCRP. REG is not a substrate for OCT1, OCT2, MATE1, and MATE2-K, but is an inhibitor of these membrane transporters [6,7,8,9,10]. OLA is a substrate for the MATE1 and MATE2-K transporters involved in its renal excretion but is not a substrate for OCT1 and OCT2. Inhibitory activity of OLA was observed on OATP1B1, OAT3, OCT1/2, MATE1, and MATE2K (IC_50_ values ranging from 5.5 to 47.1 μM) [15,22,32].

**Figure 1:**
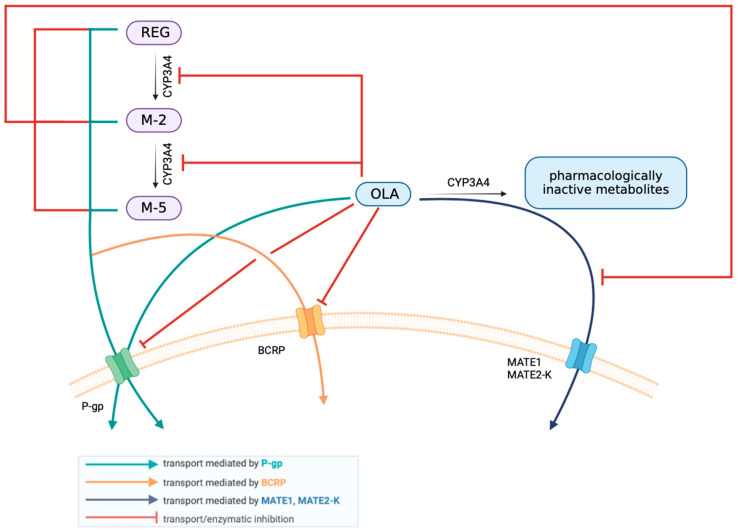
Pharmacokinetic interaction between OLA and REG.

The mechanism of pharmacokinetic interaction between OLA and REG is shown in Figure 1.

Given the literature reports on the therapeutic potential of both drugs [6,7,9,10,11,21], there is a likelihood of their combined use in the clinical setting, especially in patients with advanced gastrointestinal cancers, including CRC or HCC. However, due to the fact that the two drugs share common metabolic pathways [6,7,8,9,10,15,22,32], there is a risk of clinically relevant pharmacokinetic interaction, which could affect the final therapeutic effect or the severity of adverse effects [1,2,3,33,34,35,36,37]. In addition, REG and OLA are drugs that have only recently been introduced into the treatment of oncologic diseases, so there is insufficient clinical experience with their use.

Therefore, the proposed preclinical study to evaluate the effects of OLA on the pharmacokinetics of REG and its active metabolites M-2 and M-5, as well as of REG on the pharmacokinetics of OLA, may clarify much in this regard and guide clinical studies and observations in patients.

## 2. Materials and Methods

### 2.1. Reagents

OLA (CAS No. 763113-22-0), REG (CAS No. 755037-03-7), M-2, M-5, and REG-d3 (internal standard for the quantification of REG, M-2, and M-5) were purchased from LGC Standards Sp. z o.o. (Łomianki, Poland). OLA-d4 (internal standard for the quantification of OLA) was from LGC Standards (Toronto Research Chemicals Inc, North York, ON, Canada).

LC-MS grade methanol, acetonitrile, water, formic acid, ammonium acetate, and dimethyl sulfoxide (DMSO) were purchased from Sigma-Aldrich (Saint Louis, MI, USA). The water used in the mobile phase was deionized, distilled, and filtered through a Millipore system (Direct Q3, Millipore, Darmstadt, Germany, USA) before use. REG (Stivarga^®^, batch No. BXJRJJ1) was received from Bayer Poland Sp. z o.o (Warsaw, Poland). OLA (Lynparza^®^, batch number RR214) was purchased from AstraZeneca Pharma Poland Sp. z o.o. (Warsaw, Poland).

Drug-free rat plasma was obtained from the Anima Sp. z o. o. SK and ViVARI s.c. (Warszawa, Poland).

### 2.2. Animals

Healthy, 14-week-old adult male Wistar rats purchased from the Anima Sp. z o. o. SK and ViVARI s.c. (Warszawa, Poland) were used in the study. The animals were housed for one week in a temperature- and humidity-controlled room with a 12-h light/dark cycle and fed a standard diet with free access to fresh water.

The experiments were carried out according to the European Union Directive 2010/63/EU [38] and approved by the Local Ethics Committee (approval no 85/2022 of 16 December 2022) located in the Department of Animal Physiology and Biochemistry, Poznań University of Life Sciences, Wołyńska 35, 60-637 Poznań, Poland.

### 2.3. Study Protocol

Three groups of animals were used in the study: one study group, I_REG+OLA_ (n = 8), received REG with OLA, and two control groups, II_REG_ (n = 8) and III_OLA_ (n = 8), received REG and OLA, respectively.

As required by the Local Ethics Committee, the pharmacokinetic data from the control groups II_REG_ and the III_OLA_ were adapted from the authors’ previous projects on pharmacokinetic interactions between REG and atorvastatin [39] and OLA and metformin [40]. This reduced the number of animals included in the experiment, which is consistent with the 3R concept [41]. The same drugs (same manufacturer, same dose) were administered in the study group I_REG+OLA_, but from different batches than in the control groups. No other experimental conditions were changed.

OLA and REG were solubilized in 10% dimethyl sulfoxide (DMSO) and administered using a gastric probe (1 mL of each solution) directly into the animals’ stomachs at doses of 100 mg/kg body weight (b.w.) [40] and 20 mg/kg b.w. [39], respectively.

Blood samples in the volume of 100 µL were collected from the tail vein into heparinized tubes prior to the administration of the drug, and at 0.25, 0.5, 1, 2, 3, 5, 7, 9, 24, 26, 48, and 72 h for REG and 0.08, 0.17, 0.33, 0.50, 0.75, 1, 2, 3, 4, 6, 8, 10, 12, and 24 h for OLA. The samples were centrifuged (2880 g, 10 min at 4 °C), and the plasma was stored at −80 °C until analyzed.

### 2.4. UPLC-MS/MS Analysis of OLA, REG, M-2, and M-5

The plasma concentrations of OLA, REG, M-2, and M-5 were analyzed using the UPLC-MS/MS method [42,43] and validated according to European Medicines Agency (EMA) guidelines [44].

OLA in animal plasma samples was quantified using an Acquity 1-class PLUS ultra-high performance liquid chromatograph coupled to a Xevo TQ-S micro triple quadrupole mass spectrometer (Waters Corporation, 34 Maple Street, Milford, MA, USA) [42]. A Cortecs UPLC C18 column (2.1 × 50 mm, 1.6 µm, Waters Corporation, 34 Maple Street, Milford, MA, USA) was used for the chromatographic separation. The column temperature and injection volume were set at 40 °C and 1 µL, respectively. The mobile phase consisted of acetonitrile (eluent A) and 2 mM ammonium acetate in water (eluent B) with 0.1% 98–100% formic acid. The flow rate was kept at 0.3 mL/min. The elution gradient was as follows: 0–3 min, 5% A; 4–5 min, 95% A; 3–5 min, linear from 5% to 95% A; 5–6 min, linear from 95% to 5% A. The mass spectrometer was operated in multiple reaction monitoring mode. Two transitions were monitored for OLA and OLA-d4 as an internal standard (IS): *m*/*z* 435.1→367.1 and 435.1→281.0 (qualifier transition) for OLA and *m*/*z* 439.1→367.1 and 439.1→281.0 for IS.

The determination of REG, M-2, and M-5 was performed using an established LC-MS/MS system consisting of a Xevo TQ-S-micro triple quadrupole mass spectrometer coupled with a UPLC Acquity I-class PLUS (Waters Corporation, Milford, MA, USA), and Waters MassLynx V4.2 SCN1017 Software [43]. A Cortecs UPLC C18 column (2.1 × 50 mm, 1.6 μm) was used in combination with a VanGuard Acquity UPLC BEH C18 pre-column (2.1 × 5 mm, 3/Pk, Waters Corporation). The injection volume was 5 μL, the autosampler temperature was 4 °C, and the column temperature was 40 °C. The composition of mobile phase A was 0.1% aqueous formic acid solution (*v*/*v*), and phase B was acetonitrile/methanol and 0.1% aqueous formic acid solution in a ratio of 1:3 (*v*/*v*). The total run time was 5 min. The gradient was as follows: 0–0.5 min with 10% B; 0.5–3 min, 95% B; 3–4 min, 95% B; 4–4.1 min, 10% B; 4.1–5 min, 10% B. The flow rate was 0.4 mL/min. Multiple reaction monitoring (MRM) experiments using positive electrospray ionization (ESI) were performed to analyze the samples.

The Xevo TQ-S micro mass spectrometer (Waters) was operated in the positive ion mode and configured in the multiple reaction monitoring mode for the detection of REG, REG-N-oxide (M-2), N-desmethyl-REG-N-oxide (M-5), and isotope-labelled REG-d3 as an internal standard (IS).

The following settings of the Xevo TQ-S micro mass spectrometer were used: source temperature 150 °C, desolvation temperature 600 °C, nitrogen gas flow 900 L/h, capillary voltage 3.6 kV. Transition ion pairs (parent *m*/*z*–daughter *m*/*z*) were identified using the MRM mode for the following compounds: 482.95 to 270.08 and 288.02 for REG; 499.00 to 304.01, 252.16, and 229.00 for M2; 485.94 to 202.02 and 228.98 for M5; and 486.02 to 273.07 for REG-d3.

### 2.5. Pharmacokinetic Assay

The values of the pharmacokinetic parameters of OLA, REG, and M-2 and M-5 were determined by non-compartmental analysis with linear interpolation. PKanalix 2023R1 software (Lixoft, Antony, France) was used to calculate the elimination rate constant (k_el_), the absorption rate constant (k_a_), the half-life in the elimination phase (t_1/2_), the area under the concentration–time curve from zero to the last measurable concentration (AUC_0–t_), the area under the plasma concentration–time curve from zero to infinity (AUC_0–∞_), the apparent plasma drug clearance (Cl/F), and the apparent volume of distribution (V_d_/F). The maximum plasma concentration (C_max_) and the time to reach the C_max_ (t_max_) were obtained directly from the measured values. All these parameters were presented as arithmetic mean ± standard deviation (SD) and subjected to statistical analysis.

### 2.6. Statistical Analysis

Statistical analysis was performed using SAS software, version 9.4 (SAS Institute Inc., Cary, NC 27513, USA). The Shapiro–Wilk test was used to determine normality. Two pairs of groups were analyzed independently: I_REG+OLA_ vs. II_REG_ and I_REG+OLA_ vs. III_OLA_. The differences between the normally distributed variables were determined with the Student’s *t*-test. The non-normally distributed variables were analyzed with the Mann–Whitney test. A *p*-value of <0.05 was considered significant.

## 3. Results

### 3.1. Analytical Methods

The validation procedure followed the latest EMA [44] guidelines.

#### 3.1.1. OLA Analytical Method Validation

The calibration curves for OLA were prepared within a range of 0.025–35 µg/mL with a correlation coefficient r = 0.9957.

The lower limit of quantification (LLOQ) was determined at 0.025 µg/mL with acceptable precision and accuracy and S/N > 10.

The intra- and inter-run accuracy, determined as %bias, was ≤ 8% across three quality control (QC) levels (7.43 and −2.56, −0.48 and 5.04, −6.44 and 2.71% for 0.06, 9, and 20 µg/mL, respectively), and <20% for the LLOQ (−4.6 and 14.25%).

The intra- and inter-run precision of the assay (coefficient of variation, CV) was within 15% for the QC samples (5.33 and 9.98; 8.24 and 8.31; 11.39 and 13.50% for 0.06, 9, and 20 µg/mL, respectively), and below 20% for the LLOQ (4.23 and 17.56%).

#### 3.1.2. REG, M-2, and M-5 Analytical Method Validation

The linear correlation coefficient (r) values of the regression equations were as follows: for REG, r = 0.9980 (in the concentration range of 50–8000 ng/mL); for M-2, r = 0.9946 (in the concentration range of 25–2500 ng/mL); and for M-5, r = 0.9927 (in the concentration range of 25–175 ng/mL).

The lower limit of quantification (LLOQ) for REG, M-2, and M-5 was determined at 50, 25, and 25 ng/mL, respectively, with acceptable precision and accuracy and S/N > 10.

The intra- and inter-run precision of the assay (coefficient of variation, CV) for REG, M-2, and M-5 was within 15% for the QC samples and <20% for the LLOQ, and for REG were 5.73 and 1.93, 3.81 and −8.39, 0.23 and −2.15% for 150, 3000, and 6000 ng/mL, respectively, and 6.77 and 13.38% for 50 ng/mL; for M-2, were 3.18 and −6.8, 2.44 and 11.16, 2.57 and −5.25% for 75, 750 and 2000 ng/mL, respectively, and 0.59 and −4.8% for 25 ng/mL; for M-5, were 6.66 and 8.27, 4.06 and −3.9, 1.25 and −3.43% for 75, 100 and 150 ng/mL, and 0.26 and 8.72% for 25 ng/mL.

No carry-over effect was observed after injecting a blank sample into the LC system following analysis of the high concentration sample.

The stability of OLA, REG, M-2, and M-5 was evaluated using three concentrations of QCs. The concentrations were diluted in five replicates.

Short-term and long-term stability were tested at −20 °C storage conditions and ambient temperature at processing conditions. For the short term, three cycles of freezing and thawing within three days were applied.

The long-term test was performed after 181 days of storage for OLA and 180 days for REG, M-2, and M-5. All met ICH acceptance criteria. OLA was stable in plasma for at least 181 days and REG, M-2, and M-5 for 180 days.

### 3.2. The Influence of OLA on the Pharmacokinetics of REG, M-2, and M-5

**Figure 2:**
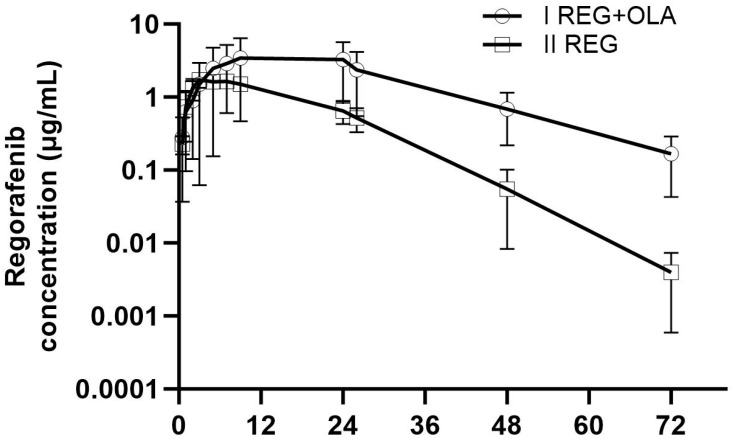
The REG plasma concentration–time profiles in rats after oral administration of a single dose of REG (20 mg/kg b.w.) to the II_REG_ group and REG + OLA (20 mg/kg b.w. + 100 mg/kg b.w.) to the I_REG+OLA_ group.

**Figure 3:**
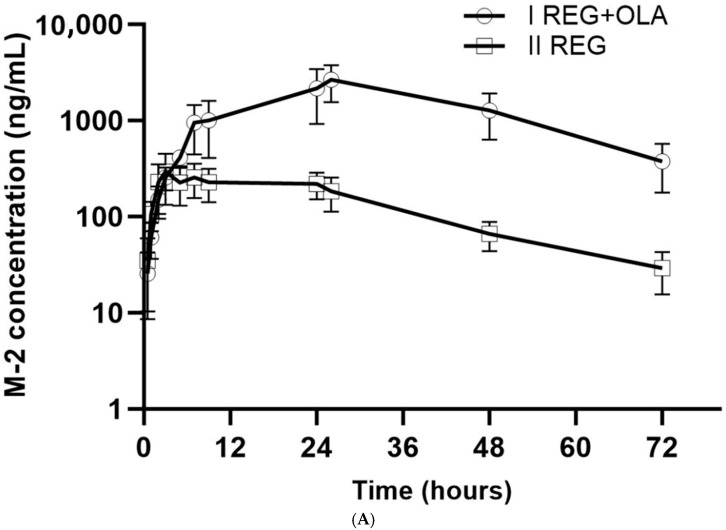

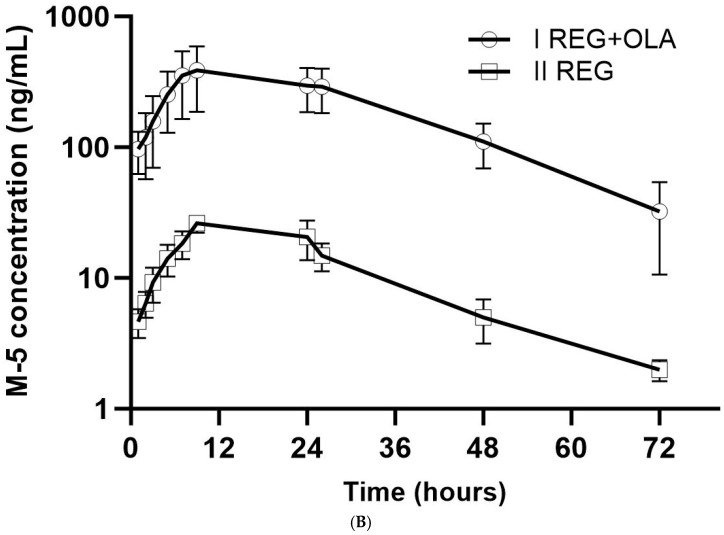
(**A**). The M-2 plasma concentration–time profiles in rats receiving REG (II_REG_) and REG+OLA (I_REG+OLA_). (**B**). The M-5 plasma concentration–time profiles in rats receiving REG (II_REG_) and REG+OLA (I_REG+OLA_).

The mean plasma concentrations of REG, M-2, and M-5 in the study and control groups are shown in Figure 2 and Figure 3A,B. The pharmacokinetic profile of REG, M-2, and M-5 was shown to be significantly altered during co-administration of OLA (Table 1). After OLA administration, the pharmacokinetic parameters of REG (AUC_0–∞_, t_max_, and t_0.5_) increased significantly by 3.38-, 2.66-, and 1.82-fold, respectively (*p*-value < 0.05). On the other hand, REG elimination parameters, i.e., k_el_ and Cl/F, were significantly reduced in the study group compared to the control group by 1.77- and 1.70-fold, respectively.

During combined administration of OLA and REG, C_max_ and AUC_0–t_ values for M-2 were 7.22 and 8.86 times higher, respectively, and for M-5, C_max_, AUC_0–t_, and AUC_0–∞_ were 16.32, 17.83, and 18.82 times higher, respectively.

The Metabolite M-2/Parent and Metabolite M-5/Parent ratios for the C_max_ and AUC_0–t_ increased by 6.52-, 10.74-, 28-, and 13-fold, respectively.

The sum of the C_max_ values for REG, M-2, and M-5 in the study group I_REG+OLA_ (5844.08 ± 3793.77 ng/mL; CV = 64.9%) was significantly greater (*p* = 0.0089) than in the control II_REG_ group (2078.95 ± 691.36 ng/mL; CV = 33.3%).

### 3.3. The Influence of REG on the Pharmacokinetics of OLA

**Figure 4:**
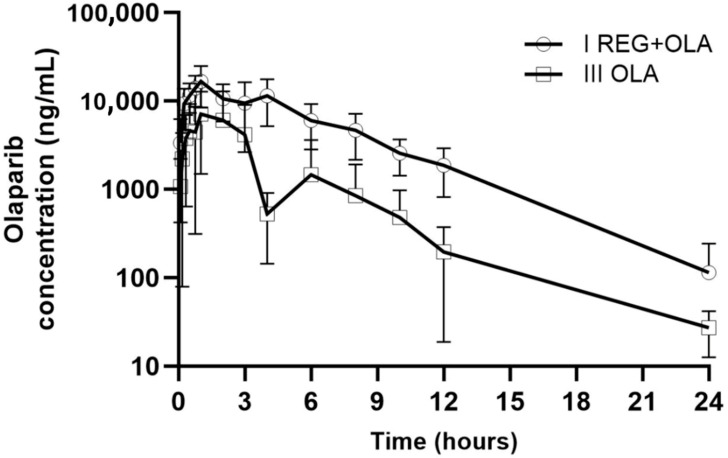
The OLA plasma concentration–time profiles in rats after oral administration of a single dose of OLA (100 mg/kg b.w.) to the III_OLA_ group and REG + OLA (20 mg/kg b.w. + 100 mg/kg b.w.) to the I_REG+OLA_ group.

The mean plasma concentration–time profiles and the pharmacokinetic parameters of OLA in the control (III_OLA_) and study (I_REG+OLA_) group are shown in Figure 4 and Table 2, respectively. After the administration of OLA with REG, the C_max_, AUC_0–t_, and AUC_0–∞_ of OLA increased 2.0, 3.4, and 3.4-fold, respectively, compared to the control group. Meanwhile, Cl/F and Vd/F of OLA in the presence of REG significantly decreased (*p* = 0.0006 and 0.0281, respectively). The value of the AUC_0–∞_ ratio (study group/control group) was 3.41.

## 4. Discussion

Data from the literature suggest that OLA may be used in combination with other anticancer drugs to treat cancers associated with different gene mutations [11,12,13,14]. For example, OLA is proving to be effective in the treatment of cancers with ATM mutations, such as colorectal cancer, for which REG is used [12,13]. OLA has also been shown to be well-tolerated and highly effective in prolonging survival in patients with advanced gastrointestinal cancers, including hepatocellular carcinoma, when used in combination with other anticancer drugs [17,18,19,20,21].

OLA and REG are metabolized by the CYP3A4 isoenzyme of cytochrome P450. Both drugs are also substrates and inhibitors of the membrane transporters P-glycoprotein and BCRP [6,7,8,9,10,15,22,32,33,36,37]. Therefore, the potential concomitant use of OLA and REG may result in clinically relevant drug–drug interactions.

Knowledge of the influence of membrane transporters and cytochrome P450 enzymes on the pharmacokinetics of drugs makes it possible to assess their impact on the efficacy and safety of therapy [45,46,47].

In this regard, the study aimed to evaluate the bilateral pharmacokinetic interactions of OLA and REG and its active metabolites after a single administration in healthy rats.

### 4.1. The Effect of OLA on the Pharmacokinetics of REG, M-2, and M-5

We found a significant 3.38-fold increase in AUC_0–∞_ (*p* = 0.0289) of REG (victim) when co-administered with OLA (perpetrator) compared to the control group. Parameters such as t_max_ (*p* = 0.0069) and t_0.5_ (*p* = 0.0003) were also significantly greater than in the control group.

However, no significant differences were observed for REG regarding C_max_, AUC_0–t_, and Vd/F in the study group compared to the control group. Co-administration of REG with OLA also affected the C_max_ and AUC_0–t_ of M-2 and M-5, which increased significantly, compared to the group of animals receiving REG alone (Table 1).

One reason for the observed increase in the AUC of REG and its active metabolites in the presence of OLA may be the inhibition by OLA of the P-gp efflux pump and BCRP in the small intestine [33,35,36].

Also, an inhibitory effect of OLA on the BCRP transporter and the CYP3A4 isoenzyme in the liver, of which REG and its active metabolites are substrates, cannot be excluded [8,32,33,35,37]. Although the inhibitory effect of OLA on P-gp and BCRP is rather insignificant for hepatic efflux, it is potentially important for intestinally expressed transporters [15].

Inhibition of the renal P-gp pump by OLA may also be the reason for the increase in plasma C_max_ of M-2 and M-5, which are P-gp substrates [8,33,35,36]. Human studies have shown that REG, as well as M-2 and M-5, tend to accumulate in the blood after administration of REG at doses above 80 mg per day [48].

The increase in exposure to REG, M-2, and M-5 can also be explained by increased absorption as seen on plasma concentration–time profiles (Figure 2 and Figure 3A,B).

OLA, by inhibiting the CYP3A4 isoenzyme in the liver, may also reduce the metabolism of M-2, resulting in an increase in its plasma concentration, as M-2 is further metabolized by CYP3A4 [48].

The value of AUC_in the presence of perpetrator_/AUC_in the absence of perpetrator_ is 3.39 and therefore seems to indicate that OLA was a moderate inhibitor in this animal model [47,49].

In contrast, the elimination process parameters of REG, i.e., k_el_ and Cl/F, were significantly reduced compared to the control group, which may indicate reduced and prolonged elimination of REG from the body.

The Metabolite M-2/Parent and Metabolite M-5/Parent ratios for the C_max_ and AUC_0–t_ increased significantly in the I_REG+OLA_ group. Also, the sum of the C_max_ values for REG, M-2, and M-5 in the study group I_REG+OLA_ (5844.08 ± 3793.77 ng/mL) was significantly greater (*p* = 0.0089) than in the control II_REG_ group (2078.95 ± 691.36 ng/mL).

The percentage of active metabolite to parent drug is important in determining dosing regimens. If it is greater than 10%, the potency of the pharmacological effect should be considered [50].

According to Steinbronn et al. [35], the recommendation to use AUC_Metabolite_/AUC_Parent drug_ ≥ 0.25 as the cutoff point for stratifying metabolite screening for CYP inhibition in vitro adequately identifies metabolites with the potential to cause DDIs in vivo. In our study, the ratio of the AUC M-2 to the AUC REG was 0.27.

When a single dose of REG is administered to humans, the peak plasma concentrations of M-2 and M-5 are much lower than those of the parent compound. However, at steady state, the concentrations of both REG and its metabolites are comparable [8].

Preclinical studies have shown that increased bioavailability of REG resulting from pharmacokinetic interactions may be associated with increased anti-tumor efficacy [51,52]. A study by Fukudo et al. [53] showed that adequate blood levels of REG, but also of its metabolites, were significant for progression-free survival (PSF) in patients with advanced metastatic colorectal cancer, gastrointestinal stromal tumor, and hepatocellular carcinoma.

Increased exposure of the body to REG may result in an increase in its pharmacological effect [51,52] but also in the occurrence of side effects, particularly in the form of liver dysfunction, rash, general weakness, the occurrence of hand-foot syndrome, or the development of hypertension [8,10].

OLA as an inhibitor of P-gp, BCRP, OATP1B1, OCT1, OCT2, OAT3, MATE1, and MATE2-K (IC_50_ values ranging from 5.5 to 47.1 μM) may interact with many drugs [15,22,32,33,36,37]. According to the Australian Public Assessment Report for Olaparib [15], OLA may alter the exposures of co-administered drugs that are substrates of CYP3A (in the intestine), P-glycoprotein (in the intestine), BCRP (in the intestine), OATP1B1, OAT3, OCT2, MATE1, and MATE2K. Regarding the ability of OLA to inhibit P-gp and BCRP transporters, the IC_50_ values are higher than 10 μM [15], which means that OLA has a low potential to inhibit P-gp and BCRP.

REG is not a substrate of OCT2, MATE1, and MATE2-K [8]. Therefore, inhibition of these transporters by OLA does not affect the pharmacokinetics of REG.

It is not yet known whether OLA will have clinically relevant DDIs when co-administered with substrates of these transporters, but this cannot be excluded. Among other things, it has been shown that caution should be exercised when OLA is co-administered with statins [54].

Zhao et al. [54] report that not only OLA but also other PARP inhibitors, i.e., niraparib, rucaparib, and veliparib, have inhibitory effects on many membrane transporters. The authors note that close monitoring for adverse effects is recommended when PARP inhibitors are administered concomitantly with some membrane transporter substrates due to the risk of clinically significant interactions.

In the authors’ previous preclinical study [40], it was shown that OLA significantly alters the pharmacokinetics of metformin. Co-administration of OLA and metformin in rats resulted in a 2.8-fold increase in metformin C_max_ and a 2.6-fold increase in AUC. This increase in metformin exposure under clinical conditions may result in a risk of adverse effects, particularly in the gastrointestinal tract [40]. As reported by McCormick et al. [32,33], OLA (17–500 μM) inhibited CYP3A4/5 with an IC_50_ of 119 μM. In time-dependent CYP inhibition assays, OLA (10 μM) had little effect on CYP3A4/5, whereas at 2–200 μM, it acted as a time-dependent CYP3A4/5 inhibitor [32,33]. As the IC_50_ for OLA is 119 µM for reversible inhibition, systemic exposure is unlikely to inhibit hepatic CYP3A activity. However, given the much higher concentrations of OLA in the gastrointestinal tract (3683 µM from a 400 mg dose) [15], inhibition of intestinal CYP3A activity is possible. CYP3A1 is the rat orthologue of CYP3A4, having 73% amino acid homology with human CYP3A4. It is also regarded as the most metabolically relevant isoform in rats [55]. The activity of CYP3A in male rats is closest to that in humans [56,57].

Therefore, clinically significant drug–drug interactions due to OLA inhibition or induction of hepatic or intestinal CYP3A4/5 cannot be excluded.

The authors concluded that OLA should be used with caution in patients with a narrow therapeutic range or sensitive CYP3A substrates.

However, Deng et al. [36] observed that OLA is a weak inhibitor of atorvastatin transport in P-gp vesicles and would have at most a minor effect on atorvastatin and rosuvastatin efflux in the small intestine.

A significant increase in t_max_ REG was seen in the presence of OLA. Both drugs, REG and OLA, are lipophilic [8,10], which means that they may compete for the rate and extent of dissolution in the gastrointestinal tract. This may have slowed the absorption of REG, in particular, which is more sensitive to pH changes. As both drugs are substrates of P-gp present in the gut [8,10], mutual competition may slow the absorption of REG, leading to a delayed achievement of maximum blood concentration. Co-administration of both drugs may also stress metabolic pathways, delaying intestinal absorption and the onset of t_max_ of REG.

### 4.2. The Effect of REG on the Pharmacokinetics of OLA

The values of OLA concentrations were higher in the study group than in the control group (Figure 4). It was also shown that REG increased the PK parameters of OLA: AUC_0–t_ and AUC_0–∞_ by 241.7% and 240.6%, respectively, and C_max_ by 104.9%.

Both OLA [15,22,32] and REG [6,7,8,9,10] are substrates of the CYP3A4 isoenzyme, so the increased bioavailability of OLA may be due to the higher affinity of REG for CYP3A4. Like REG [8], OLA is a substrate of P-glycoprotein [15].

Both drugs also have an inhibitory effect on this membrane transporter. In vitro data suggest that REG is an inhibitor of P-glycoprotein with an IC_50_ value of approximately 2 μM [10], which means that REG is a moderate inhibitor of P-glycoprotein. REG may have the potential to inhibit intestinal absorption of a co-administered P-gp substrate, as a 160 mg REG dose can be estimated to have a duodenal concentration of 1.3 mM (based on an assumed duodenal volume of 250 mL), which is significantly higher than the IC_50_ for P-gp inhibition of 2.2 µM [8,10].

The increase in OLA levels may, therefore, be due to the inhibitory effect of REG on the P-gp efflux pump in the small intestine [8,9,10]. The two drugs thus compete for both CYP3A4 affinity and affinity for membrane transporters.

REG is also an inhibitor of OCT1, OCT2, MATE1, and MATE2-K [8]. As OLA is a substrate of MATE2-K [15], the increase in its blood concentration in rats after co-administration with REG may also be due to reduced renal elimination as a result of renal inhibition of MATE1/2-K by REG. This may confirm the reduction in OLA clearance values in the study group.

In the authors’ previous study investigating the interaction of REG and atorvastatin, a significant effect of REG on the pharmacokinetics of atorvastatin metabolites, which is also a CYP3A4 substrate, was confirmed [39].

However, according to Jin et al. [37], kinase inhibitors, including REG, did not cause a significant IC_50_ shift for CYP3A inhibition. The authors found in an in vitro study that REG, masitinib, and vatalanib were competitive inhibitors of CYP3A with Ki values of 20.7, 1.3, and 0.2 μM, respectively. REG and other kinase inhibitors such as axitinib, nintedanib, tozasertib, and trametinib were predicted to cause very small AUC increases (≤1.2-fold) for (S)- and/or (R)-warfarin, suggesting a low risk of DDIs with warfarin via CYP inhibition. REG was also predicted to have little or no effect on the AUCs of apixaban and rivaroxaban. In vitro studies with human hepatic microsomes or recombinant enzymes showed that REG competitively inhibits CYP2C8, CYP2B6, CYP2C9, CYP2C19, and CYP3A4 with R1 values > 1.1; M-2 inhibits CYP2C9, CYP2C8, CYP2D6, and CYP3A4 with R1 values > 1.1 and M-5 inhibits CYP2C8 with a R1 value > 1.1. CYP3A4 contributes significantly to REG metabolism but works in conjunction with UGT1A9 [58].

The value of the AUC_0–∞_ ratio (study group/control group) was 3.41, indicating that REG was a moderate inhibitor [47] in this preclinical study.

Drug–drug interaction studies with OLA have shown that concomitant use of strong and moderate CYP3A4 inhibitors, which can significantly increase the AUC and C_max_ of this drug, should be avoided. These include itraconazole (170 and 42% increase in AUC and C_max_ of OLA, respectively) [59] or fluconazole (121 and 14% increase in AUC and C_max_ of OLA, respectively) [54]. Therefore, according to Zhao et al. [54], if a concomitant CYP3A4 inhibitor is required, the dose of OLA should be reduced to 100 mg twice daily for a strong CYP3A4 inhibitor, or to 150 mg twice daily for a moderate CYP3A4 inhibitor.

### 4.3. Summary Including Interspecies Differences

Due to interspecies differences, the translation of data from rats to humans often requires adjustments and meticulous interpretation. It is known that CYP enzymes and UGTs show significant interspecies variability, particularly in their hepatic and intestinal expression. P-gp function appears consistent across species, while BCRP and MRPs exhibit marked differences in activity and substrate specificity. These variations highlight the importance of considering species-specific characteristics when extrapolating preclinical data to humans [56,60].

Comparison of the biotransformation of REG in humans and various animal species revealed significant differences in the phase 1 and phase 2 reactions in vitro and in vivo. In humans, N-oxidation of pyridine (M-2 formation) was much more pronounced than methyl hydroxylation, both in vitro and in vivo. Glucuronidation as a primary biotransformation pathway of REG played an important role only in humans. M-2 appears to be one of the major oxidative metabolites of REG in rats. M-2 formation in the small intestine is higher in humans than in rodents. However, in all species, more M-2 was formed in the liver than in the small intestine [61].

In humans, UGT1A9 and UGT1A1 are the primary UGT enzymes involved in the glucuronidation of REG. UGT1A9 is predominantly expressed in the liver and kidney, while UGT1A1 is also present in the liver and intestine. Both enzymes contribute to the formation of REG glucuronide, the major glucuronidated metabolite in humans.

In rats, the UGT isoforms differ slightly from those in humans, with UGT1A1 and UGT2B1 being key enzymes in glucuronidation [62].

The choice of the optimal animal species to reproduce the pharmacokinetics of a drug in humans is challenging, especially in studies of DDI. The most commonly used animals are mice, rats, rabbits, dogs, monkeys, and, to a lesser extent, hamsters and guinea pigs. However, none of these models are perfect counterparts of human metabolic pathways [57,60].

However, rats are a common model for studying drug pharmacokinetics, including drug–drug interactions [63].

P-gp in rats has similar localization to human tissue, though differences in expression levels are reported. The activity of P-gp at the blood–brain barrier (BBB) is generally higher in rodents (rats and mice) compared to humans [64]. 

There are no significant species differences in P-gp function across hepatocytes between humans and rats [65].

Generally, in rats, P-gp abundance is higher in the intestine (124.1 pmol/g) compared to that in the liver (26.6 pmol/g) [66].

BCRP in rats is similarly localized, but its expression levels can vary from humans, often being lower in the intestines and kidneys. Lower BCRP levels in rat intestines lead to higher oral bioavailability of BCRP substrate drugs in rats compared to humans, while lower BCRP expression in rat kidneys results in slower renal elimination and longer systemic circulation times for these drugs in rats [67,68,69].

Absolute quantification of BCRP/Bcrp protein in the liver of different species, and the rank order as dog > rat > monkey ≈ human, was established by Li et al. [70].

Protein expression levels (fmol/cm^2^) of MDR1 and BCRP at the BBB in humans are 9.88-fold and 5.23-fold smaller than those in rats, respectively [71]. Generally, BCRP in rats is most abundant in the intestinal segments, while OATP 1a1, 1a4, 1b2, and 2a1 and MRP 2 and 6 are mainly detected in the liver [66].

Cytochrome P450 and UDP-glycosyltransferase enzymes in rats were detected mainly in the liver [66].

Cao et al. demonstrated that rats and humans show similar drug intestinal absorption profiles and transporter expression patterns in the small intestine, but distinct metabolizing enzyme expression levels and patterns make it challenging to predict human drug metabolism or oral bioavailability. The expression of CYP3A4/CYP3A9 and UDPG enzymes showed a 12- to 193-fold difference between human and rat intestine [72].

Although REG disposition varies between rats and humans due to differences in protein-binding and glucuronidation, it is significantly due to enterohepatic circulation. REG is not a substrate for OATP1 and OATP2, which means that ABCC2-mediated hepatobiliary excretion and OATP1/OATP2-mediated hepatic uptake do not play important roles in the disposition of REG [73].

Of course, the anatomical and physiological differences between rats and humans also need to be considered. In general, the anatomical structure of the gastrointestinal tract of white rats is similar to that of humans [74,75].

However, rats have relatively higher portal blood flow in relation to body weight. This may lead to faster drug metabolism and clearance from the liver. In rats, bile secretion is continuous at a constant rate due to the lack of a gallbladder. This may result in continuous recirculation of the drug, albeit at potentially lower concentrations, as bile is not concentrated in the same way as in humans. Rats have a higher activity of certain intestinal enzymes (such as β-glucuronidase) involved in the breakdown of drug conjugates excreted in the bile. This enzymatic activity allows the drug to be deconjugated and reabsorbed, improving the process of intestinal-hepatic recirculation [76].

There is a good correlation between the protein binding observed for drugs in human plasma and that for rats, although compounds tend to be slightly more bound to human plasma proteins compared to those from rats [77].

### 4.4. Limitations of the Study

The study performed has certain limitations. The differences among species regarding CYP450 should be considered. However, the activity of CYP3A in male rats is closest to that in humans, with CYP3A1, the rat orthologue of CYP3A4, exhibiting 73% amino acid homology with its human counterpart. In order to obtain reliable results and to verify the effect of the pathological condition on the pharmacokinetics of OLA and REG, a future preclinical study should be performed in animals with induced cancer. The pharmacokinetics of these drugs after repeated dosing were also not evaluated. OLA metabolites were also not determined.

## 5. Conclusions

OLA was shown to significantly affect the pharmacokinetics of REG and its active metabolites M-2 and M-5 in rats after co-administration of both drugs. There was also a significant effect of REG on the pharmacokinetics of OLA, which may have clinical relevance. The interaction observed in our study was mainly due to metabolic inhibition by the effects of both drugs on the CYP3A isoenzyme. The results obtained need to be confirmed in clinical studies. This study may provide guidance on the safety of using both drugs in clinical practice.

## Figures and Tables

**Table 1:** Pharmacokinetic parameters of REG and its metabolites M-2 and M-5 after oral administration of a single dose of REG (20 mg/kg b.w.) to the II_REG_ group and REG + OLA (20 mg/kg b.w. + 100 mg/kg b.w.) to the I_REG+OLA_ group.

Pharmacokinetic Parameters	I_REG+OLA_ (n = 8)	II_REG_ (n = 8)	*p*-Value I_REG+OLA_ vs. II_REG_	G_mean_ Ratio ** (90% CI) I_REG+OLA_ vs. II_REG_
REG				
C_max_ (µg/mL)	3.69 ± 2.97 (80.5)	1.87 ± 0.31 (16.5)	0.5054	1.37 (0.73; 2.58)
AUC_0–t_ (ng × h/mL)	106.92 ± 84.63 (79.2)	36.76 ± 5.84 (15.9)	0.0518	1.71 (0.73; 4.04)
AUC_0–∞_ (ng × h/mL)	124.47 ± 79.63 (64.0)	36.85 ± 5.83 (15.8)	0.0289	2.54 (1.38; 4.67)
t_max_ (h)	14.1 ± 8.2 (58.2)	5.8 ± 2.1 (36.9)	0.0069	2.27 (1.47; 3.51)
k_el_ (h^−1^)	0.06 ± 0.02 (26.1)	0.11 ± 0.03 (22.8)	0.0015 *	0.56 (0.44; 0.71)
t_0.5_ (h)	11.65 ± 3.70 (31.7)	6.37 ± 1.33 (20.8)	0.0003	1.79 (1.41; 2.26)
Cl/F (mL/h × kg)	129.86 ± 160.06 (123.1)	220.83 ± 29.74 (13.5)	0.0205	0.37 (0.20; 0.68)
V_d_/F (mL)	2025.57 ± 2097.73 (103.5)	2062.53 ± 615.85 (29.7)	0.3357	0.66 (0.34; 1.27)
M-2				
C_max_ (ng/mL)	2469.92 ± 1257.44 (50.9)	342.10 ± 132.87 (38.8)	0.0007	6.83 (4.52; 10.33)
AUC_0–t_ (ng × h/mL)	85,176.80 ± 35,788.00 (42.0)	9615.36 ± 2275.41 (23.7)	0.0007	8.38 (5.92; 11.87)
AUC_0–∞_ (ng × h/mL)	2469.92 ± 1257.44 (50.9)	11,469.93 ± 2133.05 (18.6)	0.0007	6.83 (4.52; 10.33)
Metabolite M-2/Parent ratio				
C_max_	1.24 ± 0.89 (71.9)	0.44 ± 0.73 (39.76)	0.0007	5.73 (3.34; 9.83)
AUC_0–t_	1.44 ± 1.09 (75.9)	0.27 ± 0.08 (29.77)	0.0007	6.39 (3.07; 13.29)
M-5				
C_max_ (ng/mL)	428.18 ± 194.48 (45.4)	26.23 ± 3.98 (15.2)	0.0007	14.23 (10.39; 19.63)
AUC_0–t_ (ng × h/mL)	14,324.20 ± 5574.43 (38.9)	803.19 ± 177.02 (22.0)	0.0007	16.13 (12.03; 21.62)
AUC_0–∞_ (ng × h/mL)	15,959.41 ± 4837.53 (30.31)	847.88 ± 178.97 (21.1)	0.0016	18.43 (14.19; 23.93)
Metabolite M-5/Parent ratio				
C_max_	0.28 ± 0.22 (78.1)	0.03 ± 0.06 (170.1)	0.0007	11.93 (5.47; 26.01)
AUC_0–t_	0.26 ± 0.17 (66.2)	0.02 ± 0.01 (23.33)	0.0007	12.29 (4.22; 35.74)
AUC_0–∞_	0.37 ± 0.19 (50.9)	0.02 ± 0.01 (22.82)	0.0040	9.61 (4.41; 20.93)

C_max_, maximum observed plasma concentration; AUC_0–t_, area under the plasma concentration–time curve from zero to the time of last measurable concentration; AUC_0–∞_, area under the plasma concentration–time curve from zero to infinity; t_max_, time to the first occurrence of C_max_; k_el_, elimination rate constant; t_0.5_, half-life in the elimination phase; Cl/F, apparent plasma drug clearance; V_d_/F, apparent volume of distribution. Arithmetic means and standard deviations (SDs) are shown with coefficients of variation CVs (%) in brackets. * Student’s *t*-test; the remaining data were analyzed using the Mann–Whitney test for non-normality. ** Geometric mean (G_mean_), ratio between the I_REG+OLA_ and II_REG_ groups (%) with a 90% confidence interval (CI) in the brackets.

**Table 2:** Pharmacokinetic parameters of OLA after oral administration of a single dose of OLA (100 mg/kg b.w.) to the III_OLA_ group and REG + OLA (20 mg/kg b.w. + 100 mg/kg b.w.) to the I_REG+OLA_ group.

Pharmacokinetic Parameters	I_REG+OLA_ (n = 8)	III_OLA_ (n = 8)	I_REG+OLA_ vs. III_OLA_ *p*-Value	G_mean_ Ratio ** (90% CI) I_REG+OLA_ vs. III_OLA_
C_max_ (µg/mL)	17.87 ± 7.66 (42.9)	8.72 ± 6.80 (78.0)	0.0241	2.86 (1.36; 6.03)
AUC_0–t_ (µg × h/mL)	96.53 ± 33.23 (34.4)	28.28 ± 28.16 (99.6)	0.0006	5.98 (2.42; 14.77)
AUC_0–∞_ (µg × h/mL)	97.14 ± 33.26 (34.2)	28.58 ± 28.31 (99.1)	0.0006	5.93 (2.41; 14.62)
k_el_ (1/h)	0.25 ± 0.06 (23.2)	0.44 ± 0.34 (77.5)	0.0583 *	0.59 (0.30; 1.13)
t_max_ (h)	1.28 ± 1.11 (86.3)	1.16 ± 0.92 (78.9)	0.9120 *	1.32 (0.62; 2.80)
t_0.5_ (h)	3.01 ± 0.86 (28.6)	4.79 ± 6.06 (126.5)	0.0650 *	1.71 (0.88; 3.30)
Cl/F (L/h)	0.43 ± 0.16 (38.3)	7.84 ± 15.21 (193.9)	0.0006 *	0.15 (0.06; 0.37)
V_d_/F (L)	1.88 ± 1.05 (55.9)	22.49 ± 24.81 (110.3)	0.0281 *	0.26 (0.10; 0.65)

C_max_, maximum observed plasma concentration; AUC_0–t_, area under the plasma concentration–time curve from zero to the time of last measurable concentration; AUC_0–∞_, area under the plasma concentration–time curve from zero to infinity; t_max_, time to the first occurrence of C_max_; k_el_, elimination rate constant; t_0.5_, half-life in the elimination phase; Cl/F, apparent plasma drug clearance; V_d_/F, apparent volume of distribution. Arithmetic means and standard deviations (SDs) are shown with coefficients of variation CVs (%) in brackets. * Mann–Whitney test for non-normality; other data were analyzed using Student’s *t*-test. ** Geometric mean (G_mean_), ratio between the I_REG+OLA_ and III_OLA_ groups (%) with a 90% confidence interval (CI) in the brackets.

## Data Availability

The data presented in this study are available on request from the corresponding author.
